# Spheroid-on-chip microfluidic technology for the evaluation of the impact of continuous flow on metastatic potential in cancer models *in vitro*

**DOI:** 10.1063/5.0061373

**Published:** 2021-08-27

**Authors:** Thomas Collins, Emily Pyne, Martin Christensen, Alexander Iles, Nicole Pamme, Isabel M. Pires

**Affiliations:** 1 Hypoxia and Tumour Microenvironment Lab, Department of Biomedical Sciences, University of Hull, Cottingham Road, Hull HU6 7RX, United Kingdom; 2 Lab-on-a-Chip Research Group, Department of Chemistry and Biochemistry, University of Hull, Cottingham Road, Hull HU6 7RX, United Kingdom

## Abstract

The majority of cancer deaths are linked to tumor spread, or metastasis, but 3D *in vitro* metastasis models relevant to the tumor microenvironment (including interstitial fluid flow) remain an area of unmet need. Microfluidics allows us to introduce controlled flow to an *in vitro* cancer model to better understand the relationship between flow and metastasis. Here, we report new hybrid spheroid-on-chip *in vitro* models for the impact of interstitial fluid flow on cancer spread. We designed a series of reusable glass microfluidic devices to contain one spheroid in a microwell under continuous perfusion culture. Spheroids derived from established cancer cell lines were perfused with complete media at a flow rate relevant to tumor interstitial fluid flow. Spheroid viability and migratory/invasive capabilities were maintained on-chip when compared to off-chip static conditions. Importantly, using flow conditions modeled *in vitro*, we are the first to report flow-induced secretion of pro-metastatic factors, in this case cytokines vascular endothelial growth factor and interleukin 6. In summary, we have developed a new, streamlined spheroid-on-chip *in vitro* model that represents a feasible *in vitro* alternative to conventional murine *in vivo* metastasis assays, including complex tumor environmental factors, such as interstitial fluid flow, extracellular matrices, and using 3D models to model nutrient and oxygen gradients. Our device, therefore, constitutes a robust alternative to *in vivo* early-metastasis models for determination of novel metastasis biomarkers as well as evaluation of therapeutically relevant molecular targets not possible in *in vivo* murine models.

## I. INTRODUCTION

*In vitro* cancer research has traditionally employed two-dimensional (2D) static methods for evaluating tumor progression leading to metastasis.^1^ However, the *in vivo* tumor microenvironment (TME) is a three-dimensional (3D) dynamic environment.^2–4^ Indeed, static 2D models have often been limiting in producing reliable, clinically relevant results.^5,6^ It is well established that the TME is a key driver of metastatic spread, including tumor hypoxia, the extracellular matrix (ECM), and interstitial fluid flow.^7–9^ Metastasis remains a critical clinical challenge, accounting for ∼90% of all cancer associated deaths.^10^ Interstitial fluid in the TME has gained interest recently for its role in permitting or even promoting metastasis.^11–13^ Thus, there is a need for better *in vitro* TME models and a strong interest in including interstitial flow to understand tumorigenesis leading to metastasis.

The use of 3D cancer models, such as spheroids, combined with microfluidics is well poised to address the relationship between the TME and interstitial fluid flow and its impact on cancer biology, including metastasis.^14,15^ Spheroids are 3D models of cancer that are formed where cell suspensions are grown in non/low adherent conditions, resulting in cells self-aggregating to form a sphere-like structure.^16^ Although it has been heavily documented that multicellular tumor spheroids can be utilized to study the invasive potential of cancer and the migratory capabilities of cancer cells,^17^ they are not standalone models because they neglect other aspects of the TME, such as interstitial fluid flow and shear stress. Therefore, it is necessary that hybrid models are used to attempt to tackle the complexity of cancer biology and metastasis. The use of microfluidics offers a method of introducing controlled flow to an *in vitro* cancer model to better understand the relationship between flow and TME progression toward metastasis.^18–21^

While there is a considerable range of microfluidic devices for spheroids, many of the reported systems have focused on spheroid generation as culturing devices, bioreactors, and/or high-throughput cytotoxicity studies.^19,22–29^ Even where a particular interest in flow or continuous perfusion culture of cancer cells exists, studies have mainly focused on drug delivery and effects in flow vs static conditions. For example, Nashimoto *et al*. developed a vascularized tumor-on-chip device to test the efficacy of drug delivery on MCF7 spheroids in static plates compared to microfluidic perfusion cultures for up to 24 h.^30^ Another study looked specifically at the effects of fluid shear stress on cancer cell motility and found significant differences in the invasive phenotypes of cells subjected to flow but did not utilize 3D spheroids as part of their experiment design.^31^ Other studies using non-cancer models have also evaluated the impact of using 3D non-cancer spheroids but did not use extracellular matrices as part of their device setup.^32^ Fewer devices have been applied for the study of early progression toward metastasis in the TME using spheroid-on-chip approaches.^33–36^ In particular, little attention has been given to evaluating how flow alone impacts the metastatic potential of 3D spheroids rather than the addition of cargo cells, drugs, or other variables. While some recent studies have started to address the need for a closer examination of interstitial fluid-like flow in the TME using 3D models, there remains a need for reproducible methods that can stratify our understanding of this relationship.^37,38^

Here, we are setting out to develop a hybrid model to attempt to tackle the complexity of cancer biology and metastasis. Microfluidic systems provide a tightly controlled microenvironment with the ability to manage and measure the input and output. We aimed to capitalize on this advantage of microfluidics rather than focus on high-throughput approaches (which have already been developed by others).^25,39–41^ We sought to develop a tightly controlled model of the TME, which also has the capacity for more complex manipulations to be integrated. This is desirable for detecting small changes, which could be indicative of how the TME is a key driver of metastasis.

The aim of this study was to address that need by developing a microfluidic device with those parameters, which would house viable spheroids for prolonged periods, with continuous perfusion of media replicating interstitial fluid flow, and to evaluate impact of flow on some key metastasis biomarkers. Here, we have successfully integrated spheroids into a reusable microfluidic device and confirmed their viability on-chip with continuous perfusion for up to 72 h. Importantly, using this device, we have recapitulated key metastatic phenotypes, such as invasion into extracellular matrices, and we are the first to show that flow conditions modeled *in vitro* induce the secretion of pro-metastatic factors.

## II. MATERIALS AND METHODS

### A. Cell culture and drug treatments

Cell lines used in this study were U-87 MG (glioblastoma) and MCF7 (breast adenocarcinoma), purchased from an authenticated source, the European Collection of Authenticated Cell Cultures (ECACC, UK). These cell lines were chosen as they represent a variety of well-established cancer spheroid models with different metastatic tropisms. Specifically, U-87 MG cells were selected as they are well characterized as robust 3D culture models for 3D invasion and migration assays.^42,43^ MCF7 cells were selected as an alternative tumor type to validate on-chip which robustly forms spheroids.^44^ Cells were cultured in high glucose DMEM (Dulbecco's Modified Eagle Medium) supplemented with 1% sodium pyruvate (Gibco), supplemented with 10% FBS (Gibco). Cells were maintained at 37 °C and 5% CO_2_ in a humidified atmosphere. Cells routinely tested negative for mycoplasma infection. Gemcitabine (Sigma, UK) stock solutions (100 mM) were prepared in sterile dH_2_O and stored at −20 °C.

### B. Spheroid formation and maintenance

Spheroids were formed and maintained as previously reported from either U-87 MG cells or MCF7 cells.^44,45^ In short, cells were seeded in ultralow adherence (ULA), round bottom, 96-well plates at different cell densities, dependent on experiment (typically either 2.5 × 10^4^ or 3.5 × 10^4^ cells per well). Once the cell suspension was added to the microwells, the plate was left undisturbed for 96 h in a 37 °C, 5% CO_2_ incubator to allow aggregation into spheroids.

### C. CytoTox-Glo toxicity assay

Media samples were collected off-chip (spheroid or cell monolayers conditioned media) and on-chip (spheroid effluent media) conditions and were stored at either 4 °C (fresh) or −20 °C (frozen). The CytoTox-Glo (Promega) protocol was followed as per manufacturer's instructions. In brief, media samples were transferred to a white 96-well plate (Nunclon). Each sample was loaded in triplicate. Assay buffer and AAF (alanyl-alanyl-phenylalanyl-aminoluciferin)-Glo luminogenic peptide substrate were mixed and added to media samples. Luminescence was measured after 15 min of room temperature incubation. As controls for the presence of cytotoxicity, a 2D cell monolayer was lysed using digitonin (20 mg ml^−1^). Luminescence values are relative to the amount of death protease activity present in the media, and luminescence a.u. (arbitrary units) measurements were normalized to effluent/conditioned media volume for each experimental condition. Cellular viability (CV) of controls was determined by subtracting the initial luminescence measurement (ILM) from final luminescent measurement (FLM).

### D. Fluorescein diacetate (FDA) and propidium iodide (PI) live–dead assay

Spheroid cell viability *in situ* was assessed using the fluorescent-based FDA–PI live–dead assay.^46^ In brief, spheroid media was removed, and spheroids were washed in 1× PBS. For the flow 1 device, spheroids were collected from experimental wells from both off-chip (96 well plate) and on-chip (flow 1 device). For the flow 2 device, staining was performed *in situ*. Spheroids were then stained using the FDA (5 mg ml^−1^)–PI (2 mg ml^−1^) solution prepared in phenol red-free Roswell Park Memorial Institute (RPMI) media (Corning). Samples were then incubated in the dark for 5 min, and the staining solution was subsequently removed. Spheroid images were acquired using an epifluorescence microscope (Zeiss, Germany).

### E. Fluorescence quantification

The fluorescence signal associated with FDA and PI staining was determined using an adapted method from those of Burgess *et al*. and McCloy *et al*.^47,48^ using ImageJ software (NIH).^49^ In brief, original gray scale images for each stain were opened in the software and the “polygon” tool was selected to draw around the spheroid or region of interest (ROI). ImageJ was then used to automatically measure the selected parameters; “area,” “integrated density,” and “mean grey value.” Background signal measurements (away from the ROI) were performed at the same size as the analyzed ROI. Where the background size could not be taken at the same size of the ROI, a sample without interference was taken and the value was divided by the area taken, before being multiplied by the total area of the ROI. Fluorescence intensity was calculated per unit area. Corrected total spheroid fluorescence (CTSF) was determined by subtracting the area of spheroid ROI background fluorescence from the integrated density measurements.

### F. Spheroid cell migration assays

For spheroid migration and invasion assays, the microwells (either flat wells on 96-well plates for static condition or on the microfluidic devices for flow) were coated with a range of hydrogels and matrices as previously described by Vinci *et al*.^42^ Specifically, Matrigel (10 mg/ml) or collagen (3 mg/ml) solutions were added directly to the microwell/device chamber and allowed to gel either at room temperature or at 37 °C. Spheroids were then placed on top of the gel/matrix layer, covered with media, and incubated for a range of time points. Plates or devices were then imaged on an inverted microscope (Zeiss, Germany) at 5× magnification.

### G. ELISA

A sandwich ELISA (enzyme-linked immunosorbent assay) for vascular endothelial growth factor (VEGF) and interleukin 6 (IL-6) (Novex, UK) was conducted to quantify secreted cytokine levels in conditioned media and effluent and previously reported.^50^ In brief, conditioned media or effluent media samples were added to the wells of the ELISA plate, and samples were processed as per manufacturer's instructions. The color signal produced by the bound enzyme cleaving the added substrate was measured using a spectrophotometer plate reader (BioTek ELx800, UK) at 450 nm. Secreted factor levels were normalized as picograms (pg) of 3.5 × 10^4^ cells.

### H. Device design and setup

**FIG. 1.**
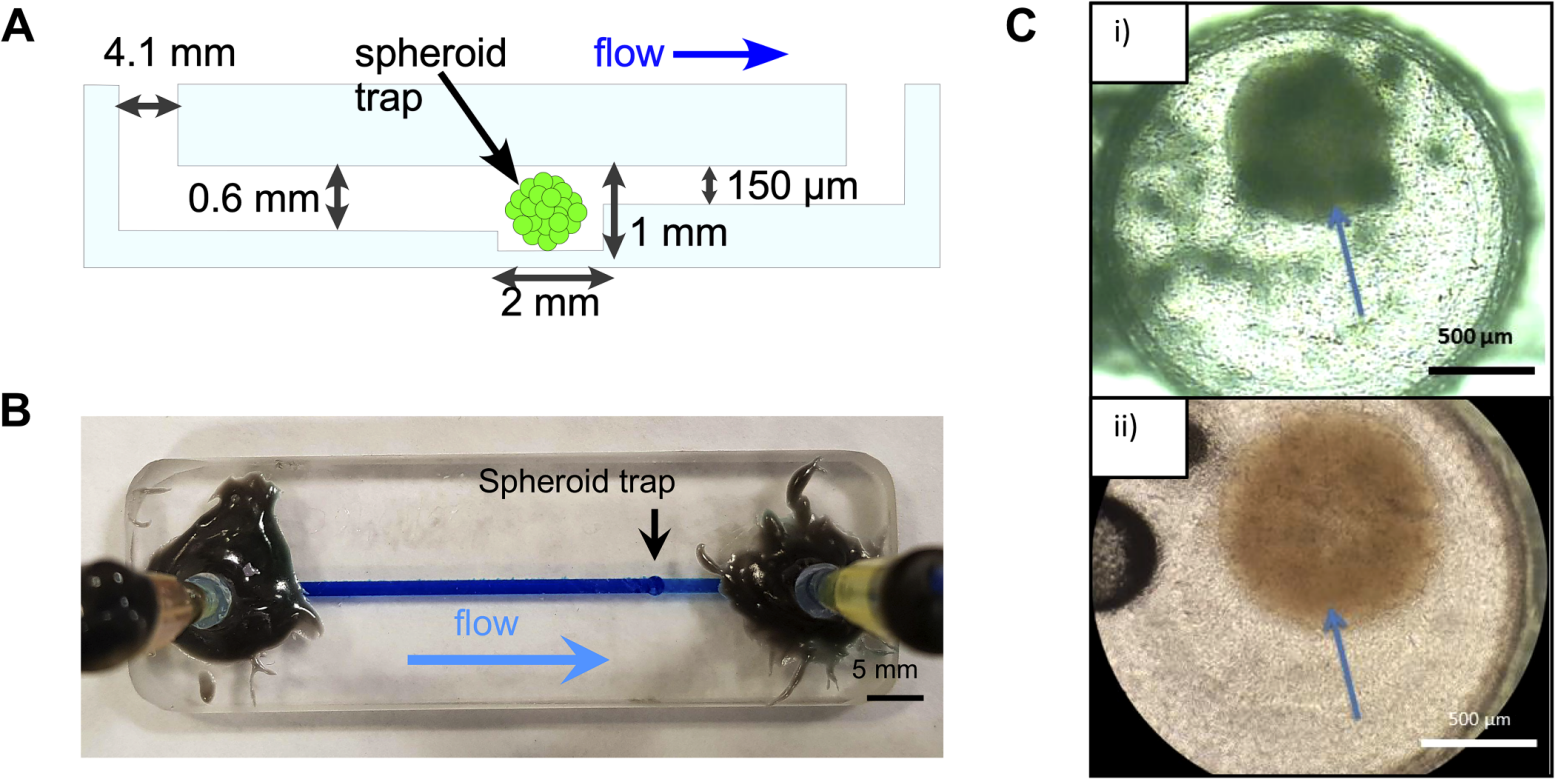
Flow 1 device design and validation. (a) Schematic side view of the flow 1 microfluidic chip showing the main components and key dimensions, as well as direction of flow. 4.1 mm wide inlet and outlet ports are placed at each end of the chip. A 1.5 mm wide channel runs from the inlet to the outlet end (55 mm long). A “trap” is situated 41.5 mm downstream from the inlet featuring a 2 mm chamber to harbor the spheroid. The channel upstream of the spheroid trap is 0.6 mm deep, and downstream of the trap it constricts to 150 *μ*m. The constriction in channel size prevents the spheroid from flowing out of the middle chamber and creates the trap. (b) Photograph of the flow 1 device fabricated from glass. The tubing for media flow is shown glued to the inlet and outlet. Brilliant blue dye flowing through the chip demonstrates the location of the channel and direction of flow. (c) Microscope images of spheroids (indicated by the arrows) inside the chamber on-chip indicating successful incorporation: (i) U-87 MG spheroid seeded at 3.5 × 10^4^ cells And (ii) U-87 MG spheroid seeded at 2.5 × 10^4^ cells.

The flow 1 device was designed to contain one spheroid in a microwell under continuous perfusion culture (Fig. 1). The devices were fabricated from two layers of Schott B270 glass of 3 mm thickness via CNC (computer numerical control) milling (Datron, Germany) using a diamond milling tool of 1 mm diameter (Eternal Tools, UK). The bottom layer featured a microwell of 2 mm diameter and 1 mm depth to hold the spheroid. This was fed by a 600 *μ*m “deep” channel with 1.5 mm width. Leading from the spheroid chamber was a 10 *μ*m “shallow” channel also of 1.5 mm width. The overall channel length, including the microwell, was 55 mm. The top layer featured 4.1 mm diameter inlet and outlet holes. The two layers were thermally bonded. Pipet tips were cut and glued with Araldite Rapid to the inlet and outlet holes and interfaced to the syringe pump (Harvard Apparatus Pump 11 Elite) with 12 cm length sections of polytetrafluoroethylene (PTFE) tubing (ColeParmer Natural, WZ-06605-27, i.d. 1.6 mm, o.d. 3.2 mm) and 1 cm length sections of larger Tygon tubing (VWR, i.d. 3.2 mm, o.d. 6.4 mm). Media were pumped at a constant flow rate of 3 *μ*l/min.

The flow 2 device (Fig. 4) was also fabricated in Schott B270 glass via CNC milling. To improve inspection via the microscope, the microwell was milled all the way through the thickness of the glass, and a microscope glass coverslip was glued to the bottom. This design also featured a 75 *μ*m height weir at the entrance of the shallow channel to minimize the potential of a spheroids moving into the outlet channel. Furthermore, a hole was also milled directly above the microwell to allow the direct pipetting of spheroids into and out of the microwell, as well as pipetting of ECM-like matrices and hydrogels into the microwell. A removable transparent polydimethylsiloxane (PDMS) plug was used to seal the microwell and prevent leakage. To minimize adherence of cells to the glass surface, the flow 2 device was silanized with octadecyltrichlorosilane (OTS) (Sigma, UK),^51^ followed by incubation with Pluronic ® F108 (Sigma, UK).^52^

### I. COMSOL simulations

Computer simulations were carried out in COMSOL Multiphysics 5.2 (COMSOL Inc.), as previously described.^53^ In brief, COMSOL was used to simulate the concentration of oxygen in spheroids in the microfluidic systems (flow 1 and 2). A flow rate of 3 *μ*l min^−1^ (as used in all flow experiments) and a necrotic threshold oxygen concentration of 1.1 *μ*mol l^−1^ were used. All simulations were performed based on zero order rate kinetics derived from colorectal adenocarcinoma spheroids.^54^

### J. Statistical analysis

At least three independent repetitions were performed per experiment. Error bars represent SEM. Statistical significance was determined by using Student’s *t*-test or two-way ANOVA (with the Tukey *post hoc* multiple comparison test), as noted. Significance was considered if p < 0.05: * p < 0.05, ** p < 0.01, and *** p < 0.001. Statistical analyses were performed using GraphPad Prism version 8 (GraphPad Software, San Diego, CA, USA).

## III. RESULTS AND DISCUSSION

### A. Flow 1 device design and validation

Our initial device design (flow 1) allowed the passage of cell culture media and spheroids of varying sizes into and out of the microfluidic device. It was envisaged to entrap spheroids within the well, while allowing media to continually perfuse over the spheroid for a prolonged period of time and effluent media to be collected for further analysis (Fig. 1). Moreover, it was desirable to have a non-gas permeable device material that would be readily machinable and reusable. Schott B270 glass was thus selected as the chip material. This is an especially prudent feature at a time when single-use plastics are not desirable.

Spheroids were introduced via the inlet port and allowed to flow toward the microwell, where they became trapped [see Fig. 1(a)], supported by the shallow outlet channel. A photograph of the devices filled with blue dye is shown in Fig. 1(b). Initial experiments were aimed at establishing that spheroids could indeed reach the microwell and be trapped, as shown in Fig. 1(c). The spheroids did not disaggregate and remained intact. Once trapped, the spheroid could be exposed to continuous flow of media, allowing investigation of the effects of flow vs static conditions on the spheroids.

At this stage, we also determined that the size of spheroids used were models with appropriate growth patterns and biological characteristics, including proliferative and hypoxic zoning, and migration/invasion patterns. Large, multicellular spheroids (>200 *μ*m) contain distinct zones (core and proliferative rim), which are most relevant to tumor conditions *in vivo*. While other spheroid-on-chip models have sacrificed spheroid size to accommodate high-throughput capacity, we styled our chip to accommodate a single, larger spheroid that models the complexities of nutrient, catabolite, and gas gradients seen in tumors.

### B. Spheroids on-chip remain viable in flow conditions (flow 1 device)

**FIG. 2.**
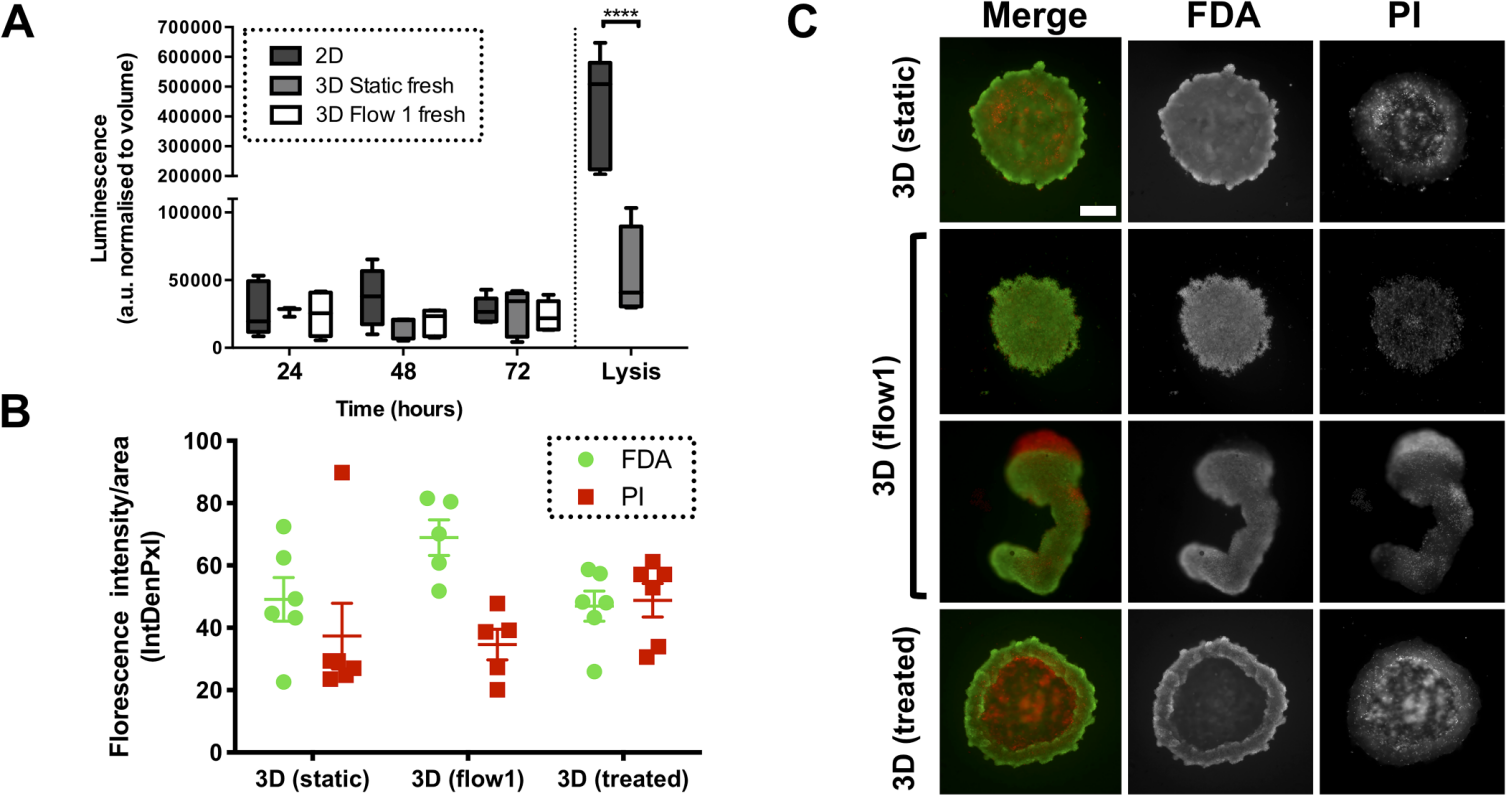
Spheroids on-chip remain viable in flow conditions (flow 1 device). (a) MCF7 spheroids were formed using 3.5 × 10^4^ cells per well as described in Sec. II. Spheroids were then either incubated in a plate in static conditions (3D static) or incorporated in the flow 1 device (3D flow 1) and exposed to flow conditions (3 *μ*l min^−1^). A 2D MCF7 culture control was also setup (2D). Samples were then incubated for 72 h, with media and effluent media samples collected at the noted time points (24, 48, and 72 h). Dead protease activity in the media, used as a surrogate for loss of cell viability, was evaluated for all samples using the using the CytoTox-Glo assay. A positive control was also established using 2D cultures lysed as per manufacturer's instructions. Luminescence a.u. (arbitrary units) measurements were normalized to effluent/conditioned media volume collect from each condition prior to the CytoTox-Glo assay being performed. The box and whiskers plot shows luminescence levels indicating dead protease activity for each sample, from three to five independent experiments. (b) and (c) MCF7 spheroids were formed using 3.5 × 10^4^ cells per well as described. Spheroids were then either incubated in a plate in static conditions (static) or incorporated in the flow 1 device (Flow 1) and exposed to flow conditions as before. As a positive control, MCF7 spheroids were treated with 100 nM gemcitabine. After 72 h, spheroids were collected from experimental wells both off-chip and on-chip, and incubated with FDA–PI live/dead stains as described in Sec. II. Spheroids in the flow 1 device were removed from the device for staining. Scatterplot (b) represents FDA (green)–PI (red) fluorescence intensity over area for all samples from three independent experiments. Raw intensity of original gray scale images of all three spheroid conditions at 72 h is plotted over area. (c) Representative FDA/PI staining images of MCF7 spheroids at 72 h. Scale bar represents 200 *μ*m. Error bars represent SEM. Two-way ANOVA was performed to test for statistical significance between samples.

Next, the spheroid viability in the flow 1 device was investigated. Microscope imaging had confirmed that MCF7 spheroids did not disaggregate in the device but could not reveal directly whether those spheroids were comprised of viable cells. It was essential that spheroids on-chip were viable to reproduce the characteristics of the TME and create an “active” 3D microenvironment. Two methods were used to determine spheroid cellular viability: CytoTox-Glo (used to measure viability markers from the effluent) and FDA/PI live/dead staining (used to determine viability *in situ*). CytoTox-Glo was chosen over the more popular LDH (Lactate dehydrogenase) assay because the latter is an end point measure and we wished to assess viability over time, at 24-h intervals. We compared the levels of protease (directly correlated with decreased viability or loss of cellular integrity) in conditioned media (2D MCF7 cell monolayers and 3D static MCF7 spheroids) and effluent samples (MCF7 spheroids on-chip under continuous perfusion of media flow conditions). There was also no significant difference in signal between fresh and frozen samples (Fig. S1 in the supplementary material). The CytoTox-Glo assay results suggested that spheroids on-chip in the flow 1 device have comparable levels of protease activity to the off-chip (static) counterparts [Fig. 2(a)].

In addition, the FDA/PI assay was used to determine *in situ* viability of MCF7 spheroids under flow conditions as compared to static conditions and treatment with 100 nM gemcitabine (used as opposite control for increased cell death). The results demonstrate that the MCF7 spheroids on-chip in the flow 1 device have a larger proportion of live cells to dead cells [Fig. 2(b)]. Although not statistically significant, there is an increase in viable cell fraction and decrease in dead cell fraction for the spheroids exposed to flow when compared to static conditions, which could be explained by the continuous perfusion of media over the spheroid and removal of nutrients and waste or improved spheroid oxygenation.^55^ It is also possible that the decreased proportion of dead cells in the spheroids on-chip is a product of the device design itself due to either dead cells becoming trapped within the internal channels or flushed out over the 72-h timecourse.^56,57^ Overall, the results did support the observations seen in the CytoTox-Glo viability assay. However, it needs to be noted that the majority of spheroids in the flow 1 device were found to be broken or misshaped. For example, in Fig. 2(c), it is evident that the spheroid has an elongated shape. In contrast, spheroids in static conditions off-chip maintain a mostly spherical structure. This could have been due to the fact that the spheroid was loaded by flowing from the inlet to the well. Also, spheroids have to be removed from the chip to allow for the FDA–PI staining. The shape alterations demonstrated that an adaptation to the device design would be needed in future iterations to maintain the ability to draw comparisons between on-chip and off-chip spheroids, so the device was redesigned to allow alternative loading and better imaging *in situ*.

### C. Revised design for integrating spheroids on-chip (flow 2 device)

**FIG. 3.**
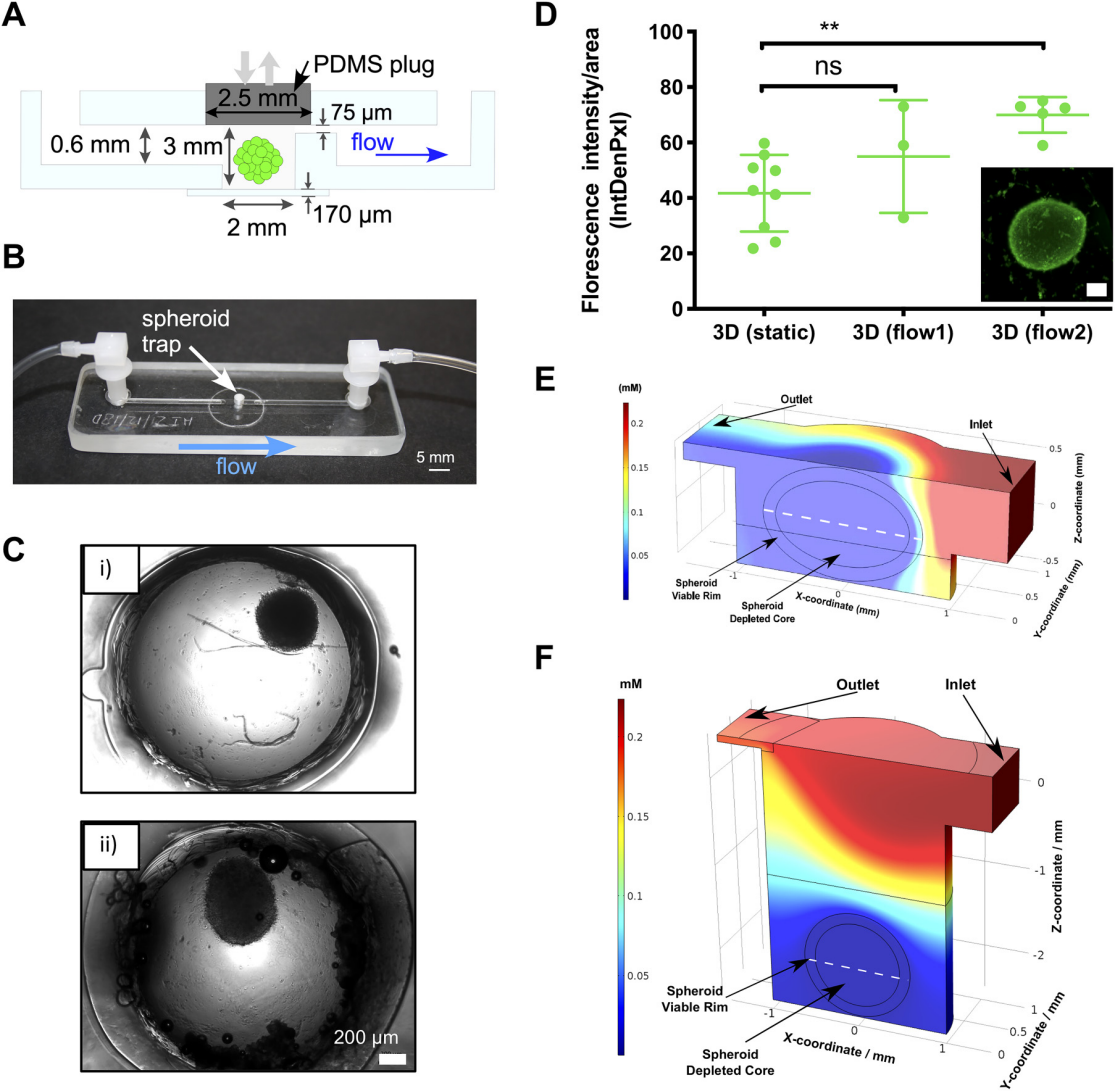
Revised design for integrating spheroids on-chip (flow 2 device). A loading port was added to the chip above the spheroid chamber. This allowed spheroids to be placed directly into the chamber rather than trapped after flowing in, as in the flow 1 device. (a) Schematic side view of the microfluidic chip showing the main components and key dimensions, as well as the direction of flow. Key differences in the flow 2 device are the loading port into the spheroid chamber, which is filled by a gas permeable PDMS “plug” during runs, and the addition of a glass coverslip, which comprises the bottom of the spheroid well and allows for clear imaging. There is also increased constriction in the channel depth following the spheroid chamber, down to 75 *μ*m. The constriction opens back up to 0.6 mm after a length of 2 mm. (b) Photograph of the flow 2 device fabricated from glass with inlet and outlet tubing attached. The direction of flow is indicated by a blue arrow. (c) Representative images of spheroids on the flow 2 device, pipetted directly into the microwell with borosilicate coverslip base for improved imaging. Images taken at 5× magnification on a Zeiss microscope under brightfield settings: (i) U87-MG spheroid (seeded at 2.5 × 10^4^ cells) in the flow 2 device and (ii) MCF7 spheroid (seeded at 2.5 × 10^4^ cells) in the flow 2 device. (d) U-87 MG spheroids were formed using 2.5 × 10^4^ cells per well as described. Spheroids were then either incubated in a plate in static conditions (static) or incorporated to either flow 1 (Flow 1) or flow 2 (Flow 2) devices and exposed to flow conditions as before. After 72 h, spheroids were collected and incubated with FDA live stain as before and imaged. FDA fluorescence intensity was quantified as before, with the scatterplot (d) representing FDA (green) fluorescence intensity over area for all samples from three independent experiments. The inset contains representative FDA staining images of U-87 MG spheroid for flow 2 at 72 h stained *in situ*. Scale bar represents 200 *μ*m. Error bars represent SEM. Student's *t*-test was used to evaluate statistical significance of fluorescence intensity changes between static conditions and each of the devices. **p < 0.01. Oxygen concentration modeling of spheroids on the flow 1 (e) and flow 2 (f) devices. Volume plot simulation of a spheroid (diameter = 1300 *μ*m) in the spheroid chamber of the flow 1 device is noted, with the spheroid perfused with air saturated medium at a flow rate of 3 *μ*l min^−1^. The mid-height cross-sectional line of the spheroid is indicated by the white dashed line. To ease the computational load, the spheroid was divided into two parts: a viable rim and a depleted core region, with higher mesh densities in the viable rim. The viable rim is 120 *μ*m thick.

**FIG. 4.**
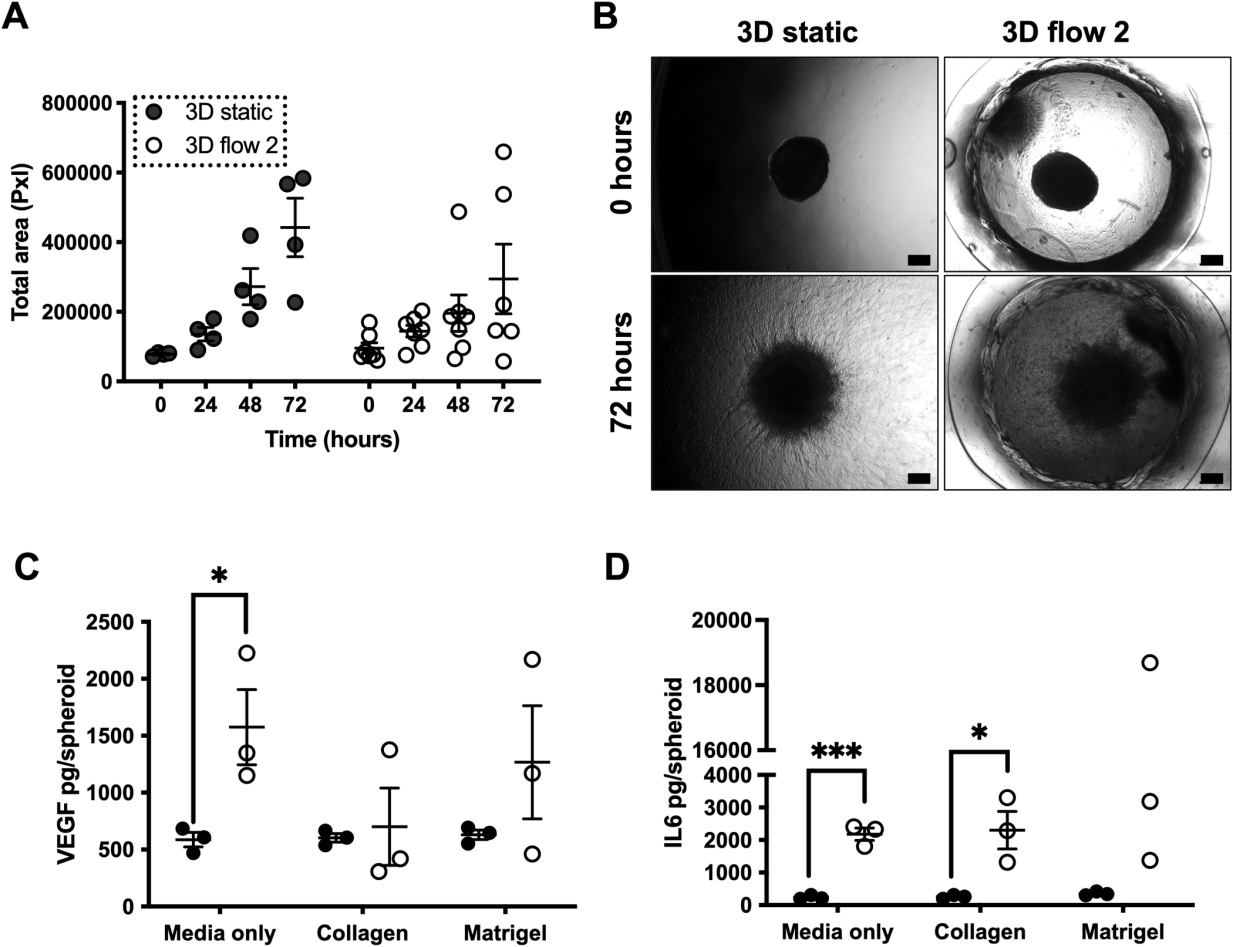
Migration and invasion capabilities of spheroids in static vs flow conditions. U-87 MG spheroids (2.5 × 10^4^ cells per well) were formed as previously described. Spheroids were then either incubated in a plate in static conditions (static) or incorporated into flow 2 (Flow 2) device and exposed to flow conditions as before. For these experiments, spheroids were incorporated into the wells or devices with varying hydrogels or media alone, as noted below. (a) and (b) U-87 MG spheroids were incorporated into wells (static) or device chamber (flow 2) containing Matrigel. Scatterplot (a) represents the total area of cellular spread from spheroid over the course of 72 h for all samples of three independent experiments. (b) Representative images of U-87 MG spheroids at 5× magnification under brightfield settings. Scale bar represents 200 *μ*m. (c) and (d) U-87 MG spheroids were incorporated into wells (static) or device chamber (flow 2) containing either media only, collagen, or Matrigel. Scatterplots represent show VEGF (c) and IL-6 (d) concentration in media/effluent samples as determined by ELISA and plotted as picograms per spheroid for three independent experiments. (a), (c), and (d) Error bars represent SEM. Two-way ANOVA was performed to test for statistical significance between samples. *p < 0.05; ***p < 0.001.

For the flow 2 design, a direct entry port into the microwell was incorporated [Figs. 3(a) and 3(b)]. This would avoid the risk of damage to spheroids as they enter the microwell via flow through an inlet channel. Instead, spheroids could now be loaded directly into the spheroid well. This also accommodated the use of larger sized spheroids; spheroids generated on-chip for high-throughput applications are generally very small,^24,40^ and we wished to continue using large spheroids to recapitulate the relevant *in vivo* zoning of tumors. Therefore, spheroids were generated off-chip with seeding at higher densities and transferred to the device after 96 h. To maintain a closed, sterile environment in the spheroid microwell, PDMS plugs were utilized to close the entry port hole after a spheroid had been loaded onto the chip [Fig. 4(a)]. A glass coverslip was incorporated at the bottom of the spheroid well to improve imaging quality, avoiding imaging through a relatively rough milled well bottom. As can be seen in Fig. 4(c), the image quality of the U-87 MG spheroids on the flow 2 device was much improved compared to the images obtained from the flow 1 device [as seen in Fig. 1(c)].

The change to the flow 2 device also improved viability of the spheroids on-chip and *in situ* FDA–PI staining. FDA staining was significantly increased in U-87 MG spheroids in the flow 2 device when compared to static conditions, indicating a clear increase in cell viability in the redesigned device [Fig. 3(d)]. We also conducted COMSOL modeling of both chips to evaluate oxygen availability [Figs. 3(e) and 3(f)]. Although a gas permeable PDMS plug was added to the flow 2 device, the deep recess of the well placed the spheroids further away from the flowing media, which is more oxygenated. This meant that there were no modeled predicted improvements in spheroid oxygenation from the flow 1 to flow 2. Although this has not seemed to impact on spheroid viability [Fig. 3(d)], this will be an important consideration for the design of further experiments using this chip as well as any designs for subsequent generations of the device.

These results demonstrated improved spheroid structure, viability, and imaging capabilities for the redesigned flow 2 device. This is particularly encouraging as it indicates the utility of conducting further morphological cell essays on-chip, such as migrating and invasion assays, that could bring further insights into the impact of flow in metastatic phenotypes.

### D. Migration and invasion capabilities of spheroids in static vs flow conditions

Once it was established that spheroids were able to be maintained viable and imaged using the flow 2 device, we evaluated whether it could be used to investigate spheroid morphological changes associated with changes in cell migration and invasion *in situ.*^38^

For this, we coated the device microwell (and corresponding 96 well plates for static controls) with biologically relevant ECM-like matrices and hydrogels and loaded U-87 MG spheroids into these conditions and incubated them for up to 72 h. U-87 MG spheroids were selected for this because they have been previously shown to be robust spheroid models for migration and invasion assays, and therefore we could verify whether our spheroids on the device were maintaining this phenotype.^42^ In order to evaluate cellular spread as a surrogate of migration/invasion capacity, we compared the total area of spread over the matrices at 24-h time points over a 72-h period [Fig. 4(a)]. The spheroids in flow conditions showed a similar, albeit not increased, area and pattern of spread than those in static conditions [Fig. 4(a)]. These experiments were conducted in Matrigel, and we speculate that either using different matrices or a combination of matrices could further influence the invasive behavior, as per previous reports.^17,58–60^ Additionally, we hope to use the flow 2 device to maintain spheroids for a longer period than 72 h and investigate whether there is a significant increase in spread after a sustained duration of continuous flow (as could be the case in the TME).

We also measured levels of pro-metastatic and pro-angiogenic cytokines VEGF and IL-6 in the effluent media of U-87 MG spheroids on-chip compared to the levels in static spheroid conditioned media. VEGF and IL-6 are cytokines that have been established as having a role in cancer progression and metastasis,^61,62^ and changes in their secreted levels could indicate early changes in the metastatic potential in a flow environment. Our results showed a significant increase in IL-6 levels in the effluent media of spheroids under flow as compared to static spheroids when cultured in media only or with collagen [Fig. 4(c)]. VEGF levels were also significantly increased in the media of flow spheroids compared to static when cultured in media only [Fig. 4(d)]. Increased levels of these cytokines in the effluent media of spheroids under flow conditions indicate that flow may indeed have an effect on cytokine expression compared to static experiments and, therefore, potentially drive metastatic potential in these conditions.

## IV. CONCLUSIONS

We designed and fabricated microfluidic chip devices to house spheroids under flow conditions to fulfill the current need for *in vitro* models, which can better recapitulate the dynamic state of the TME. Our aim was to create a device that was reusable, easily machinable, and capable of maintaining viable spheroids for a prolonged period of time. Moreover, we sought to identify the effects of continuous perfusion, or interstitial fluid-like flow, on the biology and behavior of cancer spheroids. The first design, flow 1, integrated spheroids by flowing them in through a channel and trapping them in a microwell. Spheroids were viable but the method was inconsistent for maintaining spheroid structure, and imaging was not adequate for in depth morphological analyses.

We, therefore, improved on this design by adding an access port to facilitate easy loading of spheroids directly into the well and added a glass coverslip at the bottom of the well to allow clear optical access to the spheroids maintained in the microwell. Initial investigations using this redesigned flow 2 device showed how flow can impact on the metastatic potential of these cancer 3D models, including a novel flow-mediated increase in VEGF and IL-6 cytokine levels present in the effluent media. Future work will involve further optimization of the chip design to accommodate the complexities of different metastatic tropisms as well as further investigating the signaling pathways regulated by and involved in these flow-driven changes in metastatic potential. This study highlights the need for flow to be incorporated in *in vitro* models of the impact of TME on metastatic spread.

## SUPPLEMENTARY MATERIAL

In the supplementary material, Fig. S1 shows dead protease activity measurements in the effluent is stable in fresh vs frozen effluent samples.

## AUTHORS’ CONTRIBUTIONS

T.C. and E.P. contributed equally to this work.

## Data Availability

The data that support the findings of this study are available from the corresponding authors upon reasonable request.
